# Æsthetic Surgery

**Published:** 1902-12-27

**Authors:** 


### ÆSTHETIC SURGERY.

Where the removal of the breast is necessary in consequence of the existence of malignant disease, thoroughness is of course the first consideration, but cases occur in which a wide ablation of the parts is unnecessary, and in such it sometimes becomes a matter of some æsthetic interest so to plan the operation as to leave as little trace as possible of the surgeon's knife. The scars produced by modern surgery are infinitely less conspicuous than those which were caused in pre-antiseptic times, and even than those which resulted with antiseptics during those days when prolonged drainage by india-rubber tubes was commonly employed. Still, even the suspicion of a scar across the breast is undoubtedly a "blemish"—a thing to be avoided if possible. In a recent number of *La Presse Medicale*, M. Morestin describes the method which he has adopted with the object of removing benign tumours of the breast without disfigurement. He makes a very short incision in the hollow of the axilla. He then separates the tissues by aid of a bistoury and pair of scissors, so as to form a sort of tunnel from the incision to the tumour, which in the case related was situated in the lower and outer quadrant of the breast. During this process the tumour was pushed up as high as possible towards the incision, so as to shorten the distance to be traversed, and when the tumour was reached its capsule was incised with the scissors, after which, by aid of long thin forceps, the tumour was seized and separated from its bed and thus removed. A long drain having been introduced the whole length of the "tunnel" the wound was closed. This drain was removed on the second day and the sutures on the sixth. No mark was left on the breast and the axillary scar had to be searched for before it could be discovered.

